# Robust Estimators in Geodetic Networks Based on a New Metaheuristic: Independent Vortices Search

**DOI:** 10.3390/s19204535

**Published:** 2019-10-18

**Authors:** Ismael Érique Koch, Ivandro Klein, Luiz Gonzaga, Marcelo Tomio Matsuoka, Vinicius Francisco Rofatto, Maurício Roberto Veronez

**Affiliations:** 1 Graduate Program in Applied Computing, Unisinos University, Av. Unisinos, 950, São Leopoldo 93022-000, RS, Brazil; iekoch@edu.unisinos.br (I.É.K.); veronez@unisinos.br (M.R.V.); 2 Department of Civil Construction, Federal Institute of Santa Catarina, Florianopolis 88020-300, SC, Brazil; ivandro.klein@ifsc.edu.br; 3 Graduate Program in Geodetic Sciences, Federal University of Paraná, Curitiba 81531-990, PR, Brazil; 4 Institute of Geography, Federal University of Uberlandia, Monte Carmelo 38500-000, MG, Brazil; tomio@ufu.br (M.T.M.); vinicius.rofatto@ufu.br (V.F.R.); 5 Graduate Program in Remote Sensing, Federal University of Rio Grande do Sul, Porto Alegre 91501-970, RS, Brazil

**Keywords:** geodetic networks, independent vortices search (IVS), metaheuristics, outliers detection, robust estimation

## Abstract

Geodetic networks provide accurate three-dimensional control points for mapping activities, geoinformation, and infrastructure works. Accurate computation and adjustment are necessary, as all data collection is vulnerable to outliers. Applying a Least Squares (LS) process can lead to inaccuracy over many points in such conditions. Robust Estimator (RE) methods are less sensitive to outliers and provide an alternative to conventional LS. To solve the RE functions, we propose a new metaheuristic (MH), based on the Vortex Search (IVS) algorithm, along with a novel search space definition scheme. Numerous scenarios for a Global Navigation Satellite Systems (GNSS)-based network are generated to compare and analyze the behavior of several known REs. A classic iterative RE and an LS process are also tested for comparison. We analyze the median and trim position of several estimators, in order to verify their impact on the estimates. The tests show that IVS performs better than the original algorithm; therefore, we adopted it in all subsequent RE computations. Regarding network adjustments, outcomes in the parameter estimation show that REs achieve better results in large-scale outliers’ scenarios. For detection, both LS and REs identify most outliers in schemes with large outliers.

## 1. Introduction

In 2000, the U.S. government turned off the selective availability of the Global Positioning System (GPS), making it more responsive to civil and commercial use. Since then, the industry of GNSS-based technologies (Global Navigation Satellite Systems, which includes other satellite constellations) has grown significantly. Surveyors, researchers, and most civilians use products developed from this technology. At present, GNSS receivers can be found in many devices, from precise measuring instruments to cellphones and cars. In the field of geodesy, GNSS has facilitated the implementation and quality control of high-precision and accurate networks. Before the advent of GNSS, establishing a geodetic network required a direct intervisibility between the points, as in triangulation and traverse surveys [1]. Now, point co-ordinates can be defined by GNSS signals coming from orbital space, which only requires a good coverage of satellites during data collection. This has brought great flexibility in network design, and the achievable accuracy has also helped the densification of classical first-order geodetic networks [2].

Most geodetic networks have been established using GNSS signals, which are available all over the Earth’s surface. They are defined by several materialized points with high precision and their co-ordinates serve as a basis in a wide variety of applications. Some examples are: support and basic control for surveying and mapping projects; implementation and maintenance of infrastructure works; monitoring of structural deformations; land cadastre and management; and monitoring of geodynamic events, such as earthquakes, landslides, and volcanoes. The study of Mt. Etna is a good example; two GNSS-based networks found a motion pattern of the active Nizzeti faults [3]. In addition, a geodetic network design was applied to find optimal ground locations for interferometric synthetic aperture radar (InSAR) devices [4].

To establish a geodetic network using GNSS, the receivers need to be placed at benchmarks and record satellite tracking data over a given period. Then, baseline vectors are determined between pairs of receivers, based on ranging and phase observations to the satellites. Once the vectors are computed, they become the new observations of the network and need to be adjusted to get the final point co-ordinates [1].

Although most of the GNSS observation process does not require much direct human influence, the vectors may not be free of blunders. There are sources of error that may cause significant deviations (e.g., multipath signal propagation error, cycle slips, and ionospheric anomalies). The electromagnetic wave assumed to propagate along the line of sight between the satellite and the receiver might be reflected or scattered by obstructions before reaching the instrument [5]. Other error sources are more mundane, such as mistakes in measuring the height of the antenna above the marker. Although random errors are inherent to observations, outliers should be detected, identified, and adapted for better determination of the co-ordinates [6]. An outlier is better not to be used (or not used as it is) in an adjustment process because it has a high probability of being caused by a gross error [7]. In geodesy, outliers are mostly produced by gross errors, and gross errors most often lead to outliers.

As outliers in observations affect the accuracy, those errors need to be identified or minimized [8]. In standard geodetic adjustments, a least squares (LS) process is often applied, as it is the best linear unbiased estimator, assuming that no outliers and/or systematic errors are present in observations [1,9,10]. If the data are contaminated, however, such an estimation will lead to biased parameters [11].

Alternatively, the adjustment can be based on Robust Estimators (REs), which are less sensitive to outliers. If some observations contain blunders or even systematic errors, the REs will be insensitive to those non-random errors when estimating the parameters [11]. REs have a wide range of applications; for example, in [12], a robust parameter-estimation method for a mixture model working with the weights of samples is presented. Furthermore, applications of non-Gaussian distributions on multipass SAR Interferometry have been presented in [13], and new methods for geodetic observations have been presented in [14]. Therefore, they need to be investigated for a better understanding of their capabilities and limitations in geodetic networks.

The minimizing objective functions associated with REs are not linear and, therefore, iterative processes or smart techniques are required to solve them. One approach is to combine metaheuristic algorithms (MHs), to optimize the objective function. This strategy has been applied in several studies in geodesy, as it may lead to better results than classical methods [15,16,17,18,19,20,21]. In MH research, the particle swarm optimization (PSO) [22] has been widely applied, followed by the artificial bee colony [23,24,25] and ant colony optimization methods [26], more recently [27]. Another solution is to apply a data-snooping procedure [28,29], a statistical test which takes place after the LS computation. However, studying statistical tests is not the purpose of this paper. Researchers have also applied MHs in others areas of geoscience, such as remote sensing [30,31].

For the adjustment of geodetic networks, MHs compute the unknown parameters (point co-ordinates) within a pre-defined search space and check them by evaluating the objective function for the chosen RE. The estimate is then used for the goodness-of-fitting evaluation. The process is repeated, following the exploration strategy adopted by the MH, until either an acceptable solution or the computational limit is reached [20].

Applying MHs in geodetic networks adjustment has not been widely explored. Most studies have been limited to working with only one or two REs, often in simple cases. For example, they may not have considered the generation of multiple error scenarios [18,19,21], or may have omitted the consideration of random errors [20]. It is important to explore adjustment solutions where random errors can be simulated, getting as close to a real situation as possible. Still, several error scenarios need to be tested with a variety of REs to obtain a representative set and measure the reliability of the strategy.

Furthermore, an MH needs to present stable results when minimizing the RE functions and present good strategies to avoid local minima.

Therefore, this work has two main goals: First, developing better MHs by presenting the Independent Vortices Search (IVS) based on the Vortex Search method [32], which brings advances to mitigate the limitations found in the original algorithm [33]. We compare its performance with the original method, other modifications, and a competing algorithm.

The second goal is improving quality control in geodetic networks by combining smart and robust methods. By separating the results into three scenarios (with no outliers, small outliers, and large outliers), we classify the REs according to the demand and situation.

Then, the behaviors of several known REs when adjusting a geodetic network are tested: Least Trimmed Squares (LTS) [34], Least Median of Squares (LMS) [34], LTS-RC adding a constraint [20], Sign-Constrained Robust Least Squares (SRLS) [35], Least Trimmed Absolute deviations (LTA) [36], and Iteratively Reweighted Least Squares (IRLS) [37].

We generated observations from the official co-ordinates of a station and standard deviations from GNSS signal processing. Several error scenarios were simulated in the observations, which are tested by all REs, and the results are compared with the conventional LS. In addition, different median and trim positions in residual vectors of some REs are investigated. We analyzed the results by: (1) the identification outliers, analyzing the residuals vector, and (2) by the estimated parameters, comparing them with the official co-ordinates of the network.

Additionally, for problems based on the Gauss–Markov model, we present a new proposal for the search space definition when using MHs. Furthermore, a comprehensive analysis of the results achieved with different trim and median position in some REs is given, showing which equation is best in each situation.

This paper is organized into six sections: In Section 2, we present a theoretical overview of the LS and RE methods applied in the experiments. Section 3 presents the first contribution of the study, showing the Independent Vortices Search algorithm. In Section 4, we present details of the experimental setup, how we analyzed the quality of the solutions, and the implementation of the REs and IVS. Section 5 presents the second contribution of this work, discussing the results obtained with adjustments to the GNSS network. We compare the results of the trim and median position of some REs and the outcomes, as well as discussing the various error scenarios. Section 6 brings the final considerations about the research and suggests issues to explore in future research.

## 2. Classical and Robust Approaches

The adjustment of geodetic networks is generally conducted by employing a Least Squares (LS) method, which is the best linear unbiased estimator when only random errors are present in the observations [9]. When this assumption does not comply, the estimation fails, distributing errors over many parameters. Other techniques can be applied when one suspects the presence of outliers, such as robust estimators (REs). We present a brief explanation of the methods covered in this study below.

### 2.1. Least Squares Method

The adjustment of geodetic networks applying the LS method is given by the Gauss–Markov Model [1]:

(1) Ax=l+v,

where A is the design matrix of the partial derivatives, x is the parameter correction vector (for point co-ordinates) of the linearized model, l is the observations vector minus the calculated data, and v is the residuals vector. The calculation of A results in a matrix formed by the values 0, 1, and −1, based on the partial derivatives of the observation equations:

(2) $$\begin{array}{c} \Delta X_{AB}=X_{B}-X_{A},\\ \Delta Y_{AB}=Y_{B}-Y_{A},\\ \Delta Z_{AB}=Z_{B}-Z_{A}, \end{array}$$

representing the distance between the approximate parameters on each axis.

The solution of the conventional LS adjustment is given by [9]:

(3) $$x={\left(A^{T}WA\right)}^{-1}A^{T}Wl,$$

where ^***T***^ represents the matrix transpose and ^−1^ represents matrix inversion. The quantity W is the weight matrix of the observations [1]:

(4) $$W=\sigma^{2}\Sigma^{-1},$$

where Σ is the covariance matrix of the observations and $\sigma^{2}$ is the a priori variance factor.

The final solution is given by Equation (5):

(5) $$\hat{x}=x_{0}+x,$$

where x is the parameters’ correction for the initial approximate parameter vector $\left(x_{0}\right)$, and $\hat{x}$ the vector of adjusted parameters containing the final co-ordinates of the network points.

### 2.2. Least Trimmed Squares

The Least Trimmed Squares (LTS) robust estimator consists of summing only the *h* smallest squares of the residuals (Equation (1)), suppressing the larger ones [34]. The estimator is given by:

(6) $$\min\sum_{i=1}^{h}v_{i}^{2}.$$

According to ([38] (p. 132)), *h* is defined by:

(7) $$h=\left(n+p+1\right)/2,$$

where *n* equals the number of observations and *p* is the number of parameters, which is the number of co-ordinates to be estimated.

### 2.3. Least Median of Squares

The Least Median of Squares (LMS) replaces the squared residuals summation of LS with the median square residual [34], given by:

(8) $$\min\underset{i=1,2,\ldots ,n}{{\mathrm{med}}_{h}}v_{i}^{2},$$

where the median position (med_h_) is defined by Equation (7).

### 2.4. Least Trimmed Squares with Redundancy Constraint (LTS-RC)

The LTS-RC extends LTS by adding a constraint to the summation of residuals. This estimator ensures that each parameter remains associated with at least two observations represented in the summation by their corresponding residuals. This measure guarantees observation redundancy [20].

For the adjustment of geodetic networks established only with GNSS baseline vectors as observations, the algorithm maintains at least two residuals associated with each parameter, guaranteeing redundancy. In estimation problems where a parameter depends on more than one type of observation, however, the algorithm must be adapted [39]. This is to ensure that each different type of observation is present at least twice.

### 2.5. Sign-Constrained Robust Least Squares

Unlike other REs presented so far, the Sign-Constrained Robust Least Squares (SRLS) has multiple optimization functions [35]:

(9) $$\min\left(v^{T}W_{R}v\right)/r_{W_{R}},$$

where the diagonal elements of $W_{R}$ are defined as

(10) $$w_{i}^{R}=\left\{\begin{array}{cc} w_{i}, & \;\mathrm{if}\;\left|v_{i}\right|\leq C\hat{\sigma }/\sqrt{w_{i}},\\ 0, & \;\mathrm{otherwise},\; \end{array}\right.,$$

with *C* being a positive constant defined in the range [1–2], according to the estimated number of outliers in the observations. For cases with 1% of outliers, C=2.0; for 10%, C=1.0. The quantity $\hat{\sigma }$ is a robust estimate of the standard deviation of the unit weight, in practice given by [40]:

(11) $$\hat{\sigma }=1.483\times \mathrm{med}\left(\sqrt{w_{i}}\left|v_{i}\right|\right),$$

where $\mathrm{med}\left(\sqrt{w_{i}}\left|v_{i}\right|\right)$ is the median absolute values of studentized residuals. The number $r_{W_{R}}$ is the rank of $W_{R}$.

However, due to the presence of covariance values in the weight matrix of GNSS networks, the matrix $W_{R}$ can be given by [18,37]:

(12) $$W_{R}=\mathrm{diag}\left(w_{1}^{R},\ldots ,w_{n}^{R}\right)\cdot W\cdot \mathrm{diag}\left(w_{1}^{R},\ldots ,w_{n}^{R}\right),$$

where $\mathrm{diag}\left(w_{1}^{R},\ldots ,w_{n}^{R}\right)$ are the elements calculated by Equation (10), distributed along a diagonal matrix.

In addition to the minimization of Equation (9) and the constraint for $W_{R}$, the SRLS is also subject to the following summation [35]:

(13) $$\sum_{i=1}^{n}\mathrm{sign}\left(v_{i}\right)=0.$$

### 2.6. Least Trimmed Absolute Deviations

The Least Trimmed Absolute deviations (LTA) have a formulation similar to LTS. However, the squares of the residuals are replaced by their absolute values [36]. A summation of the *h* smallest absolute residuals is carried out, ignoring the largest ones:

(14) $$\min\sum_{i=1}^{h}\left|v_{i}\right|,$$

where *h* is defined as in LTS, using Equation (7).

### 2.7. Iteratively Reweighted Least Squares

The Iteratively Reweighted Least Squares (IRLS) method represents a class of REs that works on the reweighting of W to estimate the parameters. This type of estimator has its own iterative methods to obtain a robust solution, thus not needing any external numerical method (such as MH).

The iterative method chosen was proposed in [37] and has presented good results [20,37,41].

In [37], the author presented two weight adjustment strategies. Strategy I held better results and, therefore, is adopted in this study:

(15) $$w_{i}=\left\{\begin{array}{cc} 1, & \;\mathrm{if}\;\left|\tau_{i}\right|\leq C,\\ C/\left|\tau_{i}\right|, & \;\mathrm{if}\;\left|\tau_{i}\right|>C, \end{array}\right.$$

where $\tau_{i}$ is given by

(16) $$\tau_{i}=\frac{c_{i}^{T}Wv}{\hat{\sigma }\sqrt{c_{i}^{T}WRc_{i}}},$$

with $c_{i}$ being a unit vector, only filled with a 1 in the *i*th position, and with $\hat{\sigma }$ given by Equation (11). The quantity R is the redundancy matrix [42]

(17) $$R=I-A{\left(A^{T}WA\right)}^{-1}A^{T}W,$$

where I is an identity matrix. Strategy I is, then, computed as follows [37]:

1. Initialize the counter i=0 and calculate the initial solution of the parameters ${\hat{x}}^{\left(0\right)}$ using Equation (3).
2. Increment i=i+1 and implement the weight adjustment strategy I using Equation (15).
3. Construct the equivalent weight matrix $W_{R}$ using Equation (12) by applying the calculated values to $w_{i}$.
4. Stop the iteration if the difference $∥{\hat{x}}^{\left(i\right)}-{\hat{x}}^{\left(i-1\right)}∥$ is less than a threshold value, or if a maximum number of iterations has been reached. Otherwise, return to step b and start a new iteration.

### 2.8. LMS Median Position and LTS/LTS-RC Summation Limit

As seen above, the LMS has a median position defined by *h* by applying Equation (7). Likewise, the trim limits (residuals for summation) for LTS and LTS-RC are defined, based on the same equation.

Occasionally, a variation of this equation is used [19,21,43], replacing Equation (7) by Equation (18) [38], below:

(18) $$h=\left(n+1\right)/2,$$

where *n* is the number of observations.

It is clear that the only difference, regarding Equation (7), is the absence of the variable *p* (the number of parameters). By defining *h* as above, more residuals are trimmed in LTS and LTS-RC, and the true median position of the residuals vector becomes the median value for LMS.

We can also think about other possible values for *h*. In the case of LTS and LTS-RC, a lower limit considers fewer residuals in the estimation (i.e., it will ignore more values in the residuals vector). For the LMS, the limit defines a single residual position to be taken into consideration. With a greater limit, the residual location considered the center will be further from the median, toward the larger values.

Thus, besides testing and analyzing the REs presented in this study, we also will verify the behavior of both Equations (7) and (18) for LMS, LTS, and LTS-RC estimators.

## 3. Independent Vortices Search

All of the REs (except for IRLS) require a numerical optimization method. The MH has to explore values for the parameters which generate the residuals from the Gauss–Markov model. The objective function of the RE is then evaluated and used for the computation of the solution fitness. Then, the MH generates new candidate solutions, continuing the process until it reaches a pre-defined limit [20].

For the numerical optimization, we developed the Independent Vortices Search (IVS), based on the Vortex Search algorithm (VS) [32] and its modification, the Modified Vortex Search algorithm (MVS) [33]. While VS works with one vortex, MVS can explore many vortices at once, exchanging information. We will present more details below.

### 3.1. Vortex Search Algorithm

The Vortex Search algorithm (VS) is a single solution MH for numerical function optimization. It generates new candidates around the current best result, moving along the search space when achieving a better solution. The radius defines the limit for generating new candidate solutions and decreases over cycles [32].

VS and MVS define the initial radius (radii) of the center (centers) by using Equation (19), covering the whole search space. The algorithms obtain their first solution by applying Equation (20), causing the search to begin at the center of the space:

(19) $$r_{0j}=\frac{x_{min,j}-x_{max,j}}{2},$$

(20) $$\mu_{0j}=\frac{x_{min,j}+x_{max,j}}{2},$$

where $r_{0j}$ is the initial radius of the *j*th parameter, $x_{min,j}$ and $x_{max,j}$ are the respective minimum and maximum limits of *j*th parameter, and $\mu_{0j}$ is the center of the vortex and the initial solution for the parameter *j*.

In each cycle, the radius decreases to limit the generation space for new candidate solutions. In the later cycles, the VS produces a fine adjustment as the current candidates get closer to the best solution. The radius decrease is obtained by Equation (21), and provides satisfactory control of the exploration and investigation [32]:

(21) $$r_{c}=r_{0}\cdot \left(1/R\right)\cdot \gamma \left(R,a_{c}\right),$$

where $r_{c}$ is the radius size in cycle *c*, *R* is a constant (set at 0.1) that controls the resolution of the algorithm’s search, and $\gamma \left(R,a_{c}\right)$ is the incomplete inverse gamma function, given by Equation (22):

(22) $$\gamma \left(R,a_{c}\right)=\int_{0}^{R}e^{-t}t^{a_{c}-1}dt\;a_{c}>0,$$

where $a_{c}$ is a parameter that defines the shape for each cycle, as defined by Equation (23):

(23) $$a_{c}=1-\frac{c}{C_{max}}.$$

The equation of the shape parameter uses the cycle count *c* and the maximum number of iterations $C_{max}$.

**Figure 1:**
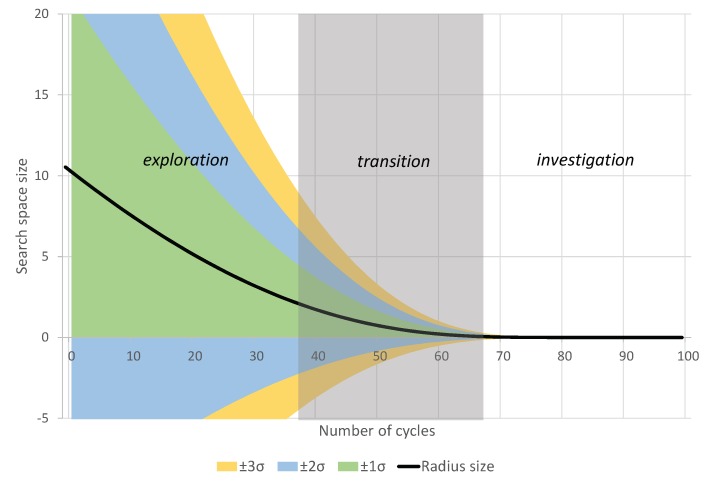
With an initial radius of size 10 and maximum cycles at 100, the black line shows the decrease of radius size, whereas the colored areas indicate the standard deviations of ±1, ±2, and ±3σ. Multivariate Normal Distribution generates about 99.7% of the candidate solutions within these intervals. At around half of the cycles, the search shifts from exploration to investigation.

The vector of candidate solutions s is randomly generated at the center μ using the Multivariate Normal Distribution (MND) with the standard deviation vector of r. Figure 1 shows an example of the radius convergence by applying Equation (21) and the candidate generation limits by the MND.

The algorithm checks that the candidate values are within the search space by applying Equation (24), where ϵ is a random real value between [0, 1]:

(24) $$x_{i,j}=\left\{\begin{array}{cc} x_{i,j}, & \mathrm{if}\;x_{min,j}\leq x_{i,j}\leq x_{max,j},\\ \epsilon \cdot \left(x_{max,j}-x_{min,j}\right)+x_{min,j}, & \mathrm{otherwise}. \end{array}\right.$$

For each candidate solution, a fitness function tests the result: If a candidate produces a better outcome than the current one, VS moves the center (μ) to the new solution and discards the remaining candidates. Otherwise, the algorithm keeps the best obtained result and produces new solutions.

Both VS and MVS use all the above equations. However, by using several vortices, MVS adjusts the positions of its centers to each cycle, based on the vortex, with a better result. This separates positioning the center $\mu_{l}$ for the generation of new solutions, and the best solution $s_{l}$ of a vortex *l*. For this, MVS applies Equation (25):

(25) $$\mu_{l}=s_{l}+\epsilon \cdot \left(s_{l}+s_{best}\right),$$

where $\mu_{l}$ is the new position of the center *l*, $s_{l}$ is the best solution of the center *l*, and $s_{best}$ is the result of the vortex with the best solution. At each cycle, all centers (except for $s_{best}$) gain new positions using the above equation [32].

As has been shown, VS has no crossover nor mutations between candidate solutions, unlike Genetic Algorithms (GAs) and the Artificial Bee Colony (ABC) method. At each cycle, it saves the best solution—either the current best or a new one—for the next cycle, discarding the remaining ones. This way, VS has no need to use individual selection strategies, a mutation rate, or other strategies present in other MHs. This helps to keep the configuration simple, reducing human interference in searching for a solution to a problem.

Another interesting characteristic of VS is the transition between global and local search. In many MHs, exploration and investigation occur from the beginning to the end of the execution. This makes it possible to find a good enough solution, even in the first few cycles. This way, they can interrupt the execution if so desired. Otherwise, the global search continues through to the last iterations, attempting to reduce the risk of local minima. In VS, the radius size is reduced over the cycles using an inverse incomplete gamma function (Equation (22)) and considering the ratio of cycles performed (Equation (21)). Thus, exploration and investigation occur gradually, and so are not present throughout the entire execution, yet still providing an adequate balance. The advantage of this strategy is the fine-tuning of the best solution found in the last cycles.

However, by analyzing the VS strategy, we can conclude that the transition between exploration and investigation has two drawbacks. First, it has the local minimum risk: There is no possibility of achieving the global solution in an execution if it limits the radius to a local minimum area. The radius of the vortex becomes small and the candidates get stuck in a local search limitation. The second inconvenience is identifying the dispensability of the processing during the execution. When defining a limit of execution for example, by the number of cycles, it executes them in its totality because only in the later cycles does VS carry out the investigation stage.

MVS tries to minimize the local problem by adding several vortices to VS. However, applying Equation (25) does not mean the vortex with the so-far best solution will bring the other centers closer to a global minimum.

With these characteristics in mind, we based the proposed IVS method on maintaining solution fine-tuning while trying to circumvent the local minimum problem, all without increasing the computational cost.

### 3.2. Characteristics of the IVS

The IVS starts from the MVS, aiming to make it simpler and more efficient. We propose three strategies through which IVS differs from MVS.

**Figure 2:**
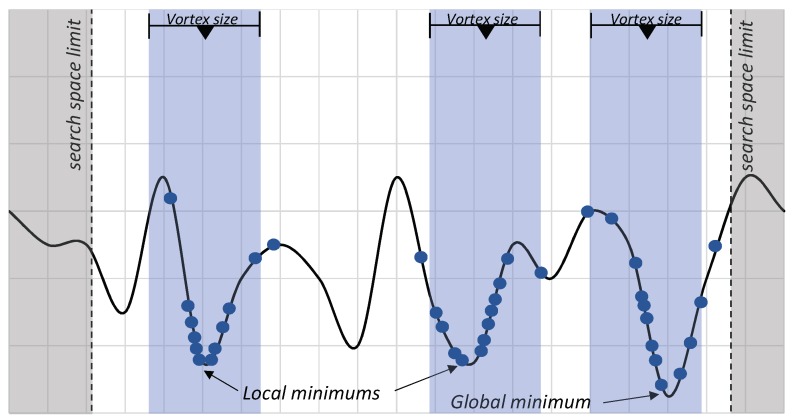
A hypothetical optimization scenario with Independent Vortices Search. The convergence of the radius of each vortex follows its investigation to a (local or global) minimum. Points represent the candidate solutions generated by each vortex.

The first major difference is the complete elimination of Equation (25), which changes the vortex center positions, based on the vortex with the best result. By doing so, each vortex can proceed toward a minimum, whether local or global. This is intended to minimize the local solutions, as we can add more vortices to explore solutions independently. Figure 2 presents a hypothetical situation in which the vortices follow their paths towards the minima.

The second feature of IVS is the replacement of Equation (20) for each additional vortex. The first vortex has an initial solution defined by applying Equation (20), whereas the remaining, by Equation (26), are randomly distributed in the search space:

(26) $$\mu_{0j}^{l}=\epsilon \cdot \left(x_{max,j}-x_{min,j}\right)+x_{min,j},$$

where $\mu_{0j}^{l}$ is the initial solution for the *j*th parameter of the vortex *l*, with l>0, $x_{min,j}$, and $x_{max,j}$ are the respective minimum and maximum limits of the *j*th parameter ,andϵ is a random real value in the interval [0, 1].

The third modification considers maintaining the amount of candidates per vortex when L>1 (where *L* is the total number of vortices). In the MVS proposal [33], the experiments presented five centers and divided the number of candidates between them. IVS, however, fixes the quantity of candidates for each vortex. To compensate for the computational increase, IVS decreases the number of cycles performed to keep the total Fitness Evaluations (FEs) the same.

It is still possible to verify that the first two strategies have the possibility of a higher diversity of the candidates generated. By removing Equation (25), each vortex is allowed to go ahead with its search, not dragging them close to a vortex, with better overall results at the moment. The second strategy (Equation (26)) allows for greater diversification while generating the first results. This is because the MND, which uses the center of the vortex as the mean for candidate generation, generates more solutions near the center. With the centers of vortices more widely distributed, IVS should not concentrate candidates in only a part of the search space.

### 3.3. A New Search Space Definition

For the definition of the search space in the adjustment of geodetic networks, we defined the limits of each parameter *j* by $x_{min,j}$ and $x_{max,j}$. By using the parameter vector x obtained from conventional LS computation, we set the exploration area according to Equation (27):

(27) $$\left\{\begin{array}{c} x_{min,j}=x_{j}-\max_{i}\left|v_{i}\right|\times \left[1-\max_{i}\mathrm{diag}{\left(R_{e}\right)}_{i}\right]\times 1.15,\\ x_{max,j}=x_{j}+\max_{i}\left|v_{i}\right|\times \left[1-\max_{i}\mathrm{diag}{\left(R_{e}\right)}_{i}\right]\times 1.15, \end{array}\right.$$

where $\max_{i}\left|v_{i}\right|$ is the greatest absolute residual from the LS solution, $\max_{i}\mathrm{diag}{\left(R_{e}\right)}_{i}$ is the greatest absorption fraction of an error and 1.15 indicates a 15% safety margin. Additionally, $R_{e}$ is defined by Equation (28) [44]:

(28) $$R_{e}=\frac{1}{\sigma^{2}}\Sigma_{\hat{v}}W,$$

where $\Sigma_{\hat{v}}$ is the covariance matrix of adjusted residuals from the LS solution.

Equation (27) presents a novel method to define the search space. It can be applied to any problem susceptible to outliers that use the Gauss–Markov model to generate residuals and MH to explore the solution. It is not limited to geodetic networks and can be used along with REs or other techniques—for example, in hyperspectral image data [45,46] or 3D Point Clouds [47]. Applying this strategy to 900 scenarios, it did not omit any solution in the IVS exploration limit.

## 4. Experimental Setup

### 4.1. The GNSS Network and Simulations

**Figure 3:**
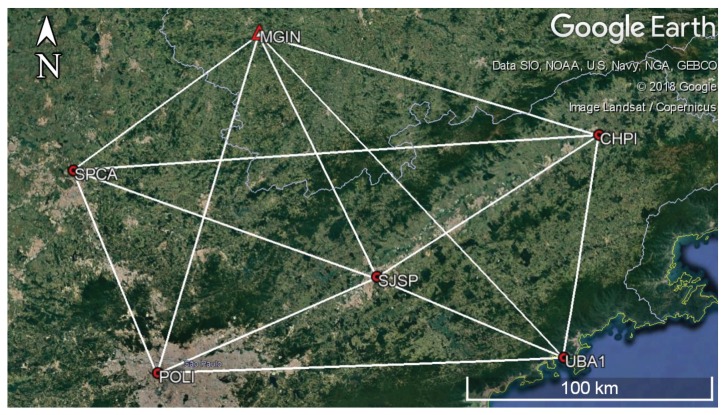
Network of six stations of the Brazilian Network for Continuous Monitoring of GNSS Systems (RBMC): One control point and thirteen three-dimensional observation vectors. Google Earth V 7.3.2.5776 (13 December 2015). Sao Paulo, Brazil. 22°57′30.85″ S, 45°55′16.44″ W, Viewpoint height 297.24 km. Image Landsat/Copernicus, Data Scripps Institution of Oceanography, National Oceanic and Atmospheric Administration, U.S. Navy, NGA, General Bathymetric Chart of the Oceans [48].

To perform the tests, we built a geodetic network from six stations in the Brazilian Network of Continuous Monitoring of Global Navigation Satellite Systems (RBMC) (Figure 3). The observation vectors were formed from the official co-ordinates of the points. We processed 6 h of data from the stations to obtain the covariance matrix ($\Sigma_{y}$). This allowed us to compute the weight matrix (W) and generate random errors for the observations. The vector l was set up by adding the official values and the random errors generated from the covariance matrix.

The network was structured without repeated observations and with independent baselines. This gives six points (one control point) and 13 vectors. As they are three-dimensional, there were a total of 15 parameters and 39 observations.

To test the RE performances, we built nine packages with 100 error scenarios each, as presented in Table 1. Package 01 had no outlier, only random errors in all observations ranging from $\left[0\sigma ,3\sigma \right)$ using a normal distribution. Trimming random errors at 3σ should not have affected the results. Following the normal distribution, the occurrence of values greater than 3σ was only 0.27%. All estimators were tested in this condition.

The other packages contained at least one outlier per scenario. Outliers were also calculated from the standard deviation of each observation. The packages separated small error scenarios (with magnitude between $\left[3\sigma ,6\sigma \right)$) from large error scenarios ($\left[6\sigma ,12\sigma \right]$). Outliers were randomly distributed over l. Packages with four outliers represented scenarios with approximately 10% contaminated observations. In the most critical cases, the network should be resistant to two simultaneous outliers between the same vertices, as we had at least four vectors connecting each station.

### 4.2. Analysis and Validation

In this work, we analyzed the quality of the solutions of each RE and the conventional LS method in three ways. First, we classified the solution regarding whether the outliers were detected or not. Second, we quantified detected, false positive, and unidentified outliers. Third, the numerical solution of the estimator was compared to the true and known co-ordinates of the points. The two first analyses were obtained using the residuals vector v, while the numerical comparison was extracted from the estimated co-ordinates vector $\hat{x}$.

We divide the classification into six classes, represented by capital letters from “A” to “F”:

- A: All outliers detected, no false positive;
- B: All outliers detected, with at least one false positive;
- C: Some outliers detected, no false positive;
- D: Some outliers detected, with at least one false positive;
- E: No outlier detected, no false positive; and
- F: No outlier detected, with at least one false positive.

We considered false positive to be a value bigger than 3σ in the residuals vector in a position where no outlier was inserted, as we truncated the random errors by up to three sigma.

Comparison of the estimated solution with the true co-ordinates was carried out in order verify the impact of the errors in the final solution of the adjusted parameters ($\hat{x}$). This allows us to consider, for example, whether an estimator was better for outlier detection or parameter estimation.

### 4.3. Implementation

#### 4.3.1. IVS Parameters

The parameters of IVS were the same for all scenarios and REs. A total of 50 candidates per vortex and 40 vortices were used, as this configuration gave the best results in our experiments. To limit the algorithm execution time, we defined the amount of fitness evaluations (FE) to 25,000,000.

The fitness evaluation of the RE solutions was conducted by Equation (29) [20]:

(29) $$fit=\frac{1}{1+|f|},$$

where *f* is the result of the objective function.

#### 4.3.2. Robust Estimators Adaptation

In LS, the weight matrix of the observations (W) plays an important role for the estimation; therefore, it is also necessary to consider it here. In GNSS networks specifically, observations are correlated, rendering the problem even more complex [41]. These observations form a weight matrix with some negative values of covariance. Hence, in LTS, the summation of the residuals has to be performed after extracting the absolute values of the weighted squared residuals, in order to eliminate the influence of the signs in the ranking. This results in the following Equation (30):

(30) $$\min\sum_{i=1}^{h}w_{i},$$

where $w_{i}$ is defined by

(31) $$w_{i}=\left|\langle W_{\sigma_{v}\left(i\right)},v^{2}\rangle \right|,\;\mathrm{for}\;i=1,\ldots ,n,$$

with $\sigma_{v}$ representing a permutation of the indices i=1,…,n, such that $0\leq w_{1}\leq w_{2}\leq \cdots \leq w_{n}$. The quantity $v^{2}$ represents the vector that has, as components, the squares of the residuals in v. The inner product is, then, given by

(32) $$\left|\langle W_{i},v^{2}\rangle \right|=\left|W_{i,1}\times v_{1}^{2}+W_{i,2}\times v_{2}^{2}+\cdots +W_{i,n}\times v_{n}^{2}\right|=\left|\sum_{j=1}^{n}W_{i,j}v_{j}^{2}\right|,$$

where $W_{i}=\left(W_{i,1},W_{i,2},\ldots ,W_{i,n}\right)$ denote the respective rows of W.

Following this idea, for LMS, the mathematical model already adapted to GNSS networks is given by:

(33) $$\min\underset{i=1,2,\ldots ,n}{{\mathrm{med}}_{h}}w_{i},$$

where $w_{i}$ is given by Equation (31), and the median position (med_h_) is defined by Equation (7).

Likewise, the LTA adapted for GNSS networks is given by:

(34) $$\min\sum_{i=1}^{h}z_{i},$$

with $z_{i}$ following the same idea as the LTS, except that v is not squared:

(35) $$z_{i}=\left|\langle W_{\sigma_{v}\left(i\right)},v\rangle \right|,\;fori=1,\ldots ,n.$$

For the SRLS, Equation (13) presents a constraint that needs to be implemented. In this study, we adopted a penalty function that multiplies the result of Equation (9) given by Equation (36):

(36) $$f_{p}=f\times \left(1+\left|\sum_{i=1}^{n}\mathrm{sign}\left(v_{i}\right)\right|\right),$$

where $f_{p}$ is the penalized result for the SRLS estimation. If Equation (13) equals 0, *f* is multiplied by 1, and the result remains unchanged. Otherwise, it will be penalized. In the sign function, absolute values smaller than $1\times 10^{-9}$ were considered as 0.

#### 4.3.3. Flowchart

**Figure 4:**
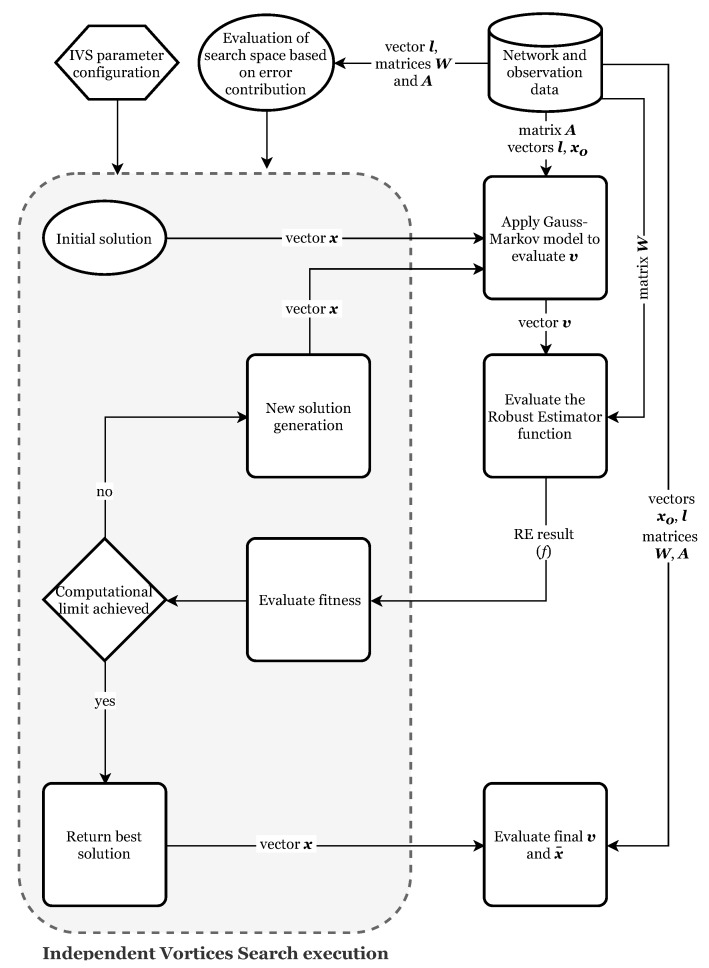
Flowchart of the network adjustment based on Independent Vortices Search and a robust estimator function. The search space is evaluated from the error contribution.

The development and implementation of the adjustment routine using REs and IVS followed the flowchart shown in Figure 4.

## 5. Results and Discussion

For better comprehension, this section is divided into three subsections: Section 5.1 presents the optimization with IVS and comparison with the performances of other MHs. Section 5.2 shows the estimated impact of the trim limit and median position in some REs, when applied to the geodetic network adjustment. Section 5.3 presents the results for the network adjustments, applying IVS and testing all the REs (including the trim limit and median position variations) in 900 error scenarios. The adjustments were organized in three topics: no outlier case, small magnitude outliers, and large magnitude outliers.

### 5.1. Performance and Discussion of IVS

We conducted several tests to compare the solutions obtained with IVS to solutions obtained by other MHs. For a better analysis, we generated four scenarios of errors in a GNSS network. Each scenario contained three simultaneous outliers, ranging from $\left[3\sigma ,6\sigma \right)$, in 39 observations (about 7.8% contamination). Using the LMS estimator, the MHs executed the scenarios twenty-five times. This allowed for analyzing the stability and quality of the results. More details of the network can be found in the next section.

Besides the MHs already presented, we also tested the Hybrid Vortex Search algorithm (HVS). The HVS works with the combination of ABC and VS, trying to take advantage of the best characteristics of each strategy. The reader can find more details in [49]. Thus, the algorithms tested were:

- Artificial Bee Colony (ABC) [23,24];
- Vortex Search algorithm (VS) [32];
- Modified Vortex Search algorithm (MVS) [33];
- Hybrid Vortex Search algorithm (HVS) [49]; and,
- Independent Vortices Search (IVS).

The configurations of the MHs obeyed the following parameters: 5,000,000 FE as processing limit, 50 candidates per center for vortex algorithms, and 50 bees in ABC (25 food sources). In addition, we tested the MVS and IVS with five vortices, as presented in [33], and 40 vortices. Preliminary tests showed better results from 20 to 60 vortices, so we adopted an intermediate value of 40.

It is important to note that cycle counting was not used as a configuration parameter or as a limit for the MH executions. For more objective comparisons between MHs, we should not use the cycle count as a stopping criterion. As each MH has different strategies in its execution, it can use a different quantity of FE in each cycle. Using a fixed number of cycles as a parameter to limit the execution can cause a large variation in the total amount of FE for each MH. This would favor MHs that make more FE per cycle, since they have more opportunities to test their solutions [50]. We adapted the algorithms to use the number of FE as the limit for suspension. This allowed for a more adequate performance comparison. To better situate the reader, executing 5,000,000 FE was equal to 100,000 cycles in ABC with 50 bees (25 food sources); 100,000 cycles in VS with 50 candidates; and 20,000 cycles in IVS with five vortices and 50 candidates per center.

The MVS experiments performed in [33] divided the quantity of candidates by the number of vortices. Thus, in executions with 50 candidates and five vortices, MVS generated only 10 candidates per vortex every cycle. As this work uses the number of FE as the execution limit of the MHs, each vortex generated a number of candidates established by the number of candidates parameter. For a run with 50 candidates and five vortices, the algorithm generated 50 candidates per vortex each cycle. This characteristic is standard in IVS.

**Figure 5:**
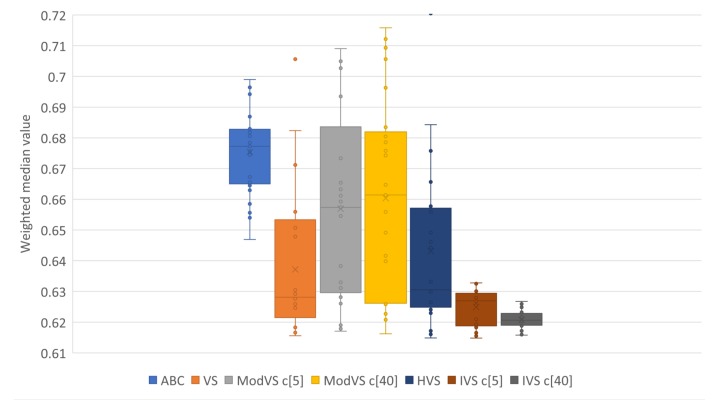
Results of 20 runs in error scenario 01 for each metaheuristic (MH). c[X] indicates the number of vortices, where applicable.

**Figure 6:**
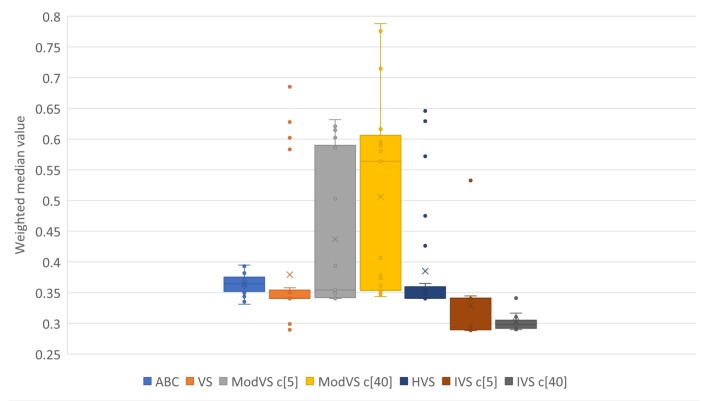
Results of 20 runs in error scenario 02 for each MH. c[X] indicates the number of vortices, where applicable.

**Figure 7:**
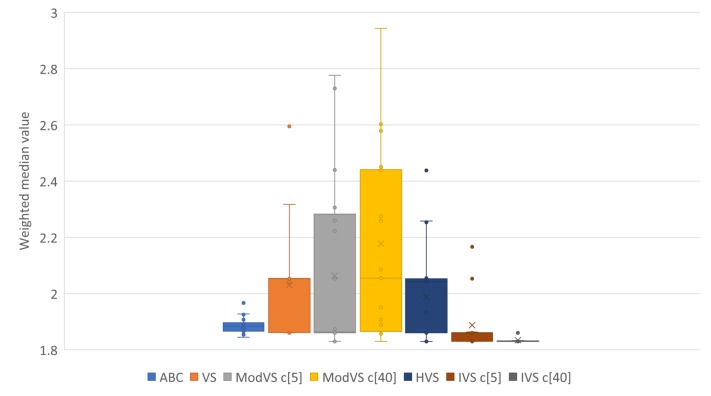
Results of 20 runs in error scenario 03 for each MH. c[X] indicates the number of vortices, where applicable.

**Figure 8:**
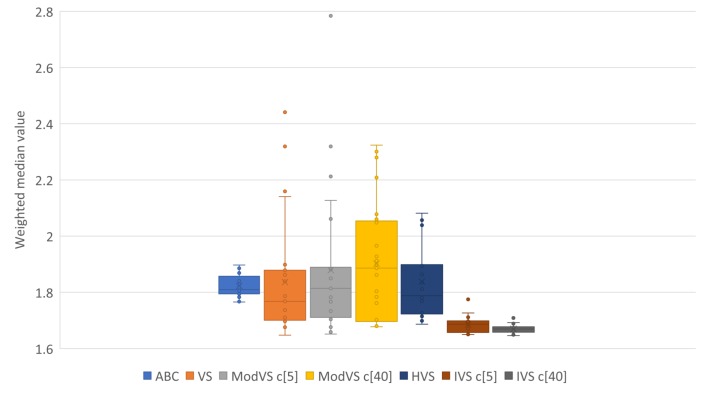
Results of 20 runs in error scenario 04 for each MH. c[X] indicates the number of vortices, where applicable.

For better representation of results, we present them in box plots. Each point represents the result of one run. The number after “c” in brackets (e.g., “c[5]” and “c[40]”) is the amount of vortices, where applicable. Figure 5, Figure 6, Figure 7 and Figure 8 show the results for Scenarios 01, 02, 03, and 04, respectively.

By analyzing the charts, we see that IVS performed better than other MHs. The configuration with 40 vortices overcame the alternative with five vortices, although both options showed good results. IVS achieved the lowest minimum and presented little variation. The HVS strategy did not improve much on the solutions, as they were similiar to those found by the original VS. ABC showed lower variation in the results when compared to the already known MHs. This shows a certain stability in the solutions, surpassed only by IVS.

It is also possible to note the horizontal alignments of points (solutions) in the charts. This was common to several MHs and identifies the local minimums in which the MHs got stuck in, on some runs. In Figure 7, it is possible to notice an alignment between the values 2,4 and 2,6 for MVS and HVS. The same diagram shows another alignment between 1.8 and 2.0 for all MHs, which coincided with the bottom of the boxes for the VS, MVS, and HVS solutions.

The MH proposed in this work overcame all other tested MH in both the mean and variation of the solution. It needed no extra computation, producing the first scientific contribution of this work. We applied the IVS configuration with 40 vortices in the following experiments to optimize the RE functions.

### 5.2. Comparing LTS/LTS-RC Trim Limit and LMS Median Position

We performed tests with the two values for the limits of LTS and LTS-RC and for the median position of LMS. The lower limit, $h=\left(n+1\right)/2$, will be shown as $h_{n2}$ and the upper limit, $h=\left(n+p+1\right)/2$, as $h_{np2}$.

**Figure 9:**
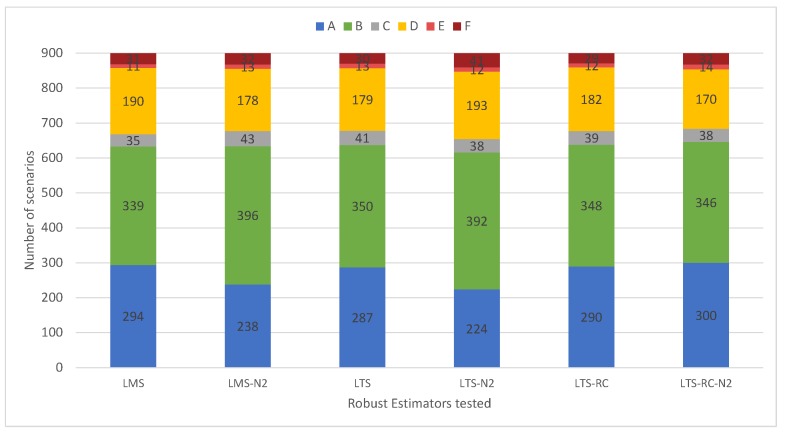
Occurrence of each classification in the 900 scenarios for the three estimators and their variation in the trim/median position. The suffix “-N2” shows the limit of $h_{n2}$, for the respective estimator. Non-suffixed REs show the limit of $h_{np2}$.

Figure 9 shows that the adjustments with the limit of $h_{n2}$ had worse results in the classification for LMS and LTS. Classifications of type “A” were reduced by adopting the expected threshold, as compared to more robust, increased “B” classifications. For these estimators, the lower limit maintained outliers identification, but pointed out more false positives. The results for the LTS-RC present a slight improvement to the limit of $h_{n2}$.

Comparing the results of LTS and LTS-RC, we notice that the results presented almost the same classifications, whereas the variations with $h_{n2}$ presented a significant difference. This is because the larger limit ($h_{np2}$) removed fewer residuals, hardly breaking the redundancy. Furthermore, since the network had several observations among its three-dimensional points (although not repeated), it was hard for any parameter to remain without at least two residuals (not breaking redundancy). Using the limit of $h_{n2}$, the classic LTS more easily broke the redundancy in the residuals trim, whereas the LTS-RC achieved more satisfactory results, retaining the redundancy of the network.

**Figure 10:**
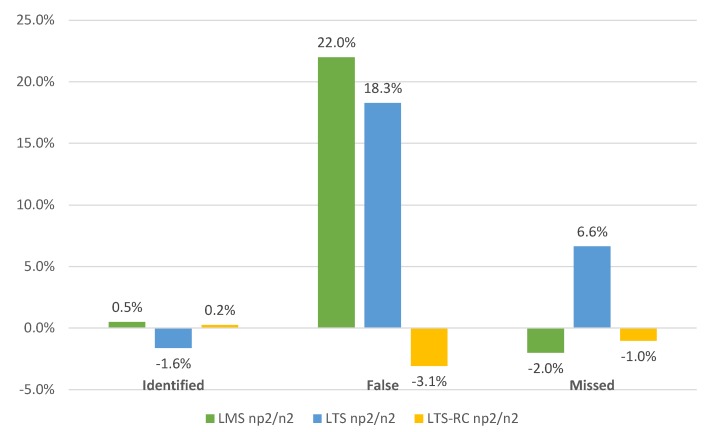
Percent difference for each estimator divided into identified errors (false and undetected) for the limits of $h_{n2}$ and $h_{np2}$. A positive percentage value means higher occurrence with $h_{n2}$.

Figure 10 confirms a gain of false positives with the limit of $h_{n2}$ for LMS and LTS, and stability with a slight improvement for LTS-RC. There were no significant differences in outlier identification for the different limits, while, for undetected errors, only the LTS showed a 6.6% worsening.

**Figure 11:**
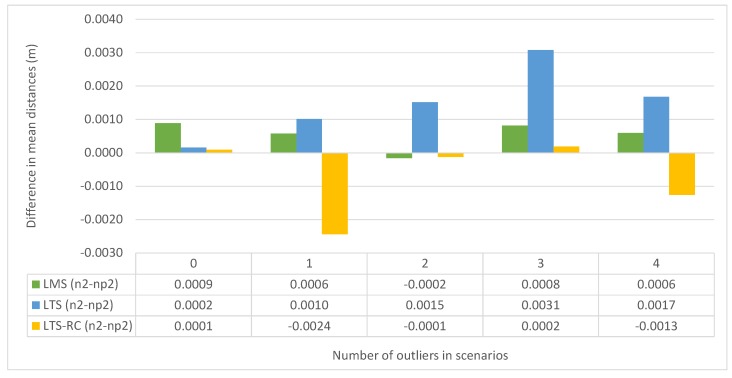
Mean distance difference of the co-ordinates from their official values. The limits $h_{n2}$ and $h_{np2}$ divided by scenario packages from 0 to 4 simultaneous outliers. Negative values mean smaller deviations with the limit of $h_{n2}$.

We also compared the distance of the co-ordinates from their true values. Figure 11 displays the mean distance difference of the co-ordinates in each scenario package, between the limits $h_{n2}$ and $h_{np2}$, for each RE. The mean differences were negligible in most situations, being below 1 mm with either $h_{n2}$ or $h_{np2}$. LTS produced inferior results with $h_{n2}$, whereas the LTS-RC reduced the variation in two cases, with one and four simultaneous outliers.

The redundancy constraint has proven to be interesting for any problems where the number of observations per parameter is more limited and/or the trim limit is smaller. To the best of our knowledge, this is the first time the *h* value has been analyzed for a geodetic application. As the measurements of these networks do not contain data related to all the parameters, the redundancy constraint also played an important role. Besides this specific treatment, the *h* value can be considered in any studies that wish to apply LMS, LTS, or LTS-RC.

### 5.3. Results in Network Adjustments

For better analysis of the behavior of each RE, we divided the results into three topics: the no outlier case (Package 01); scenarios with small outliers (Packages 02, 04, 06, and 08); and scenarios with outliers of great magnitude (Packages 03, 05, 07, and 09).

#### 5.3.1. No Outlier Case

For scenario package 01, the results confirmed the statements in the literature. The conventional Least Squares method (LS) was the best linear unbiased estimator when the observations were free of outliers.

**Figure 12:**
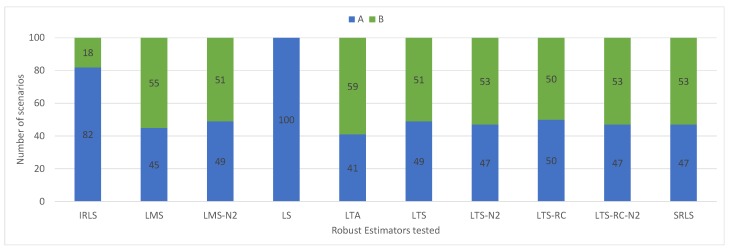
Classifications achieved in scenarios without outliers by estimator. “A” stands for no false positive, and “B” one or more false positive(s).

The solution classifications by detecting outliers in the residuals are shown in Figure 12. LS had excellent performance, with no false positives, getting an “A” classification in all scenarios. Most REs showed similar results, with about half of the classifications as “A”.

**Figure 13:**
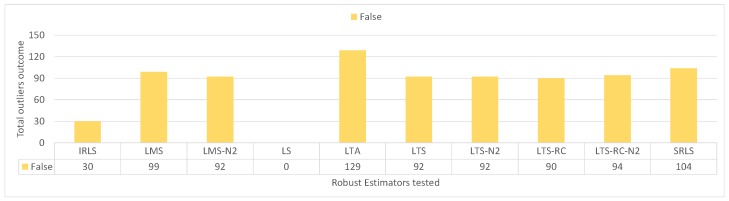
Number of false positives for the scenarios without outliers, grouped by estimator.

This means that all REs presented false positives in some scenarios. Figure 13 shows the false positive counts for each estimator. Most estimators achieved similar results, with almost one false positive per scenario.

**Figure 14:**
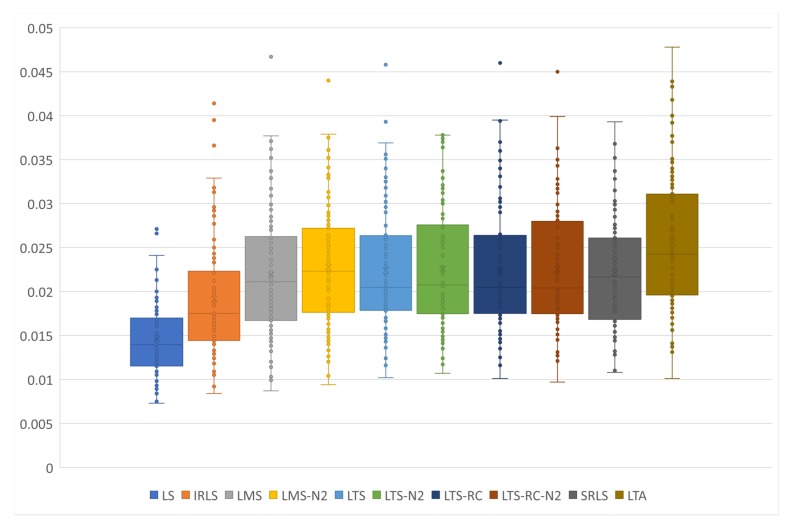
Scenarios without outliers organized by estimator. Each point represents the average absolute distance from the estimated co-ordinates of each scenario, compared to the official values of the stations in the network.

To check the solutions of each estimator, we elaborated the box-plot chart shown in Figure 14. The LS exceeded all other estimators, showing a lower variance and a lower variation between the scenarios. As expected, the LS estimation was superior to any other RE, when tested in the conditions of no outliers. In addition, LS required no MH to estimate the parameters and presented low computational cost, when compared with estimators that need MHs. It is important to point out that the points will not reach zero, due to the random errors present in all observation vectors.

Both for outlier identification and co-ordinate estimation, LS proved to be the best method for scenarios with no outliers. The high number of false positives in the other estimators solutions led to discarding good observations and greater deviations in the estimation.

#### 5.3.2. Small Magnitude Outliers

**Figure 15:**
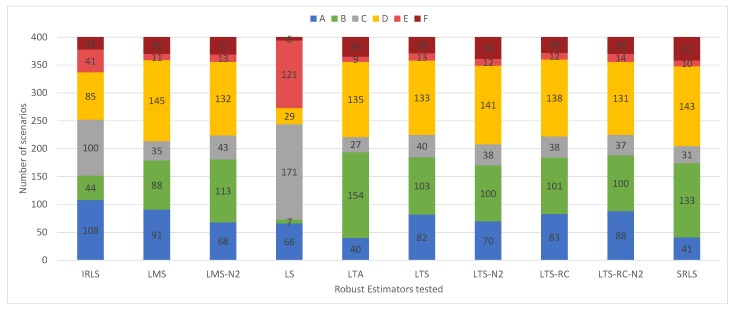
Solution classifications, from “A” to “F”, for the 400 scenarios containing 1–4 outliers of smaller magnitude, organized by estimator.

Starting with the outlier detection, Figure 15 gives the solution classifications for each RE in the 400 scenarios. LS presents its results concentrated in the ‘no false positives’ classifications (“A”, “C” and “E”). This shows a resistance for pointing out false positives in the residuals vector by LS solutions. The IRLS got the highest number of solutions classified as “A”, overcoming the other estimators. By checking the results for classifications that identified all outliers (“A” + “B”), the LMS, LTS, and LTS-RC-N2 had the best identification ability.

**Figure 16:**
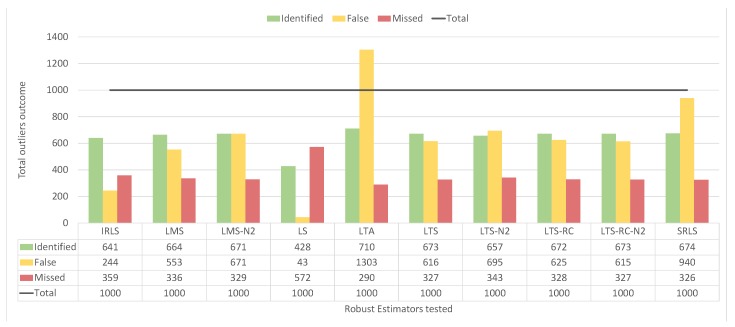
Number of identified false positives and undetected outliers for each estimator in the 400 scenarios with outliers of smaller magnitude.

Although the LTA presented many classifications of type “B”, this estimator had the lowest number of solutions classified as type “A”, similar to SRLS. In Figure 16, we see that LTA presented 1303 false positives in the residuals vector, over the 400 scenarios. It also confirms LS as the estimator with the least amount of false positives (43). In contrast, LS missed most of the blunders, not detecting 572 out of 1000 inserted outliers. Among the REs where IVS was applied, the LMS identified a good part of the outliers without an exaggerated quantity of false positives, missing less than IRLS and LS.

Analyzing the estimator solutions for parameter estimation, Table 2 shows the mean distance of solutions from the official co-ordinates. It also adds the LS result without outliers, to compare the influence of the random errors.

LS presented the smallest deviations, being the best estimate of the parameters in the case of adopting the solution, without eliminating contaminated observations and making new adjustments. Even in scenarios with four outliers, the LS showed a better result than the REs, although the difference became smaller. The IRLS presented low variation in the solutions, even with an increase of blunders. Whereas most RE presented similar solutions, LTA, in contrast, had the worst estimates.

For an application where outliers should be identified, the LS method is not the best. In these cases, according to the results of the experiments, it is recommended to work with IRLS, or, for a better identification with a higher cost in false positives, LMS, LTS, and LTS-RC-N2 obtained more satisfying results.

By analyzing the estimates, LS presented a more solid estimate, even with the lowest detection of blunders. This shows that the outliers which were not detected by LS did not exert great distortion in the estimation. For both scenarios without outliers or schemes that present blunders of small magnitude, LS remains as the best estimator for the parameters. The good redundancy of the network probably contributed to this result. In networks with poorer geometry, the LS will not be as robust to small outliers.

#### 5.3.3. Large Magnitude Outliers

**Figure 17:**
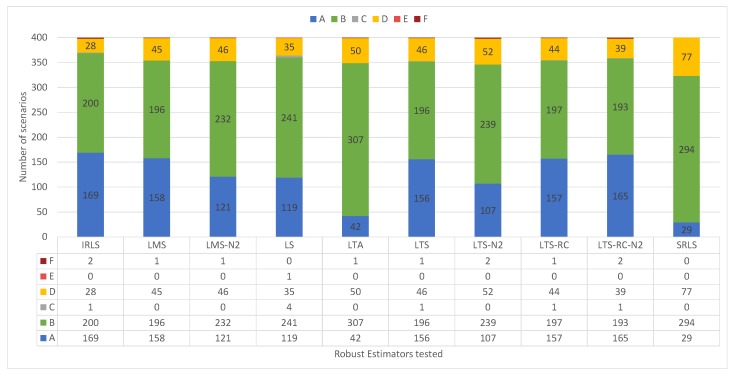
Solution classifications, from “A” to “F”, for the 400 scenarios containing 1–4 outliers of larger magnitude, organized by estimator.

In contrast to the experiments with small outliers, all REs showed greater ease in detecting larger outliers. Most the results were classified as “A’’ or “B’’, and rarely as “E’’ or “F’’ (see the classifications of the solutions in Figure 17). In this case, LS presented solutions with false positives, “B’’ and “D’’ types, resembling the other tested estimators.

For classifications “A’’and “B’’ (i.e., all outliers were identified), the estimators presented similar results, around 90%.

**Figure 18:**
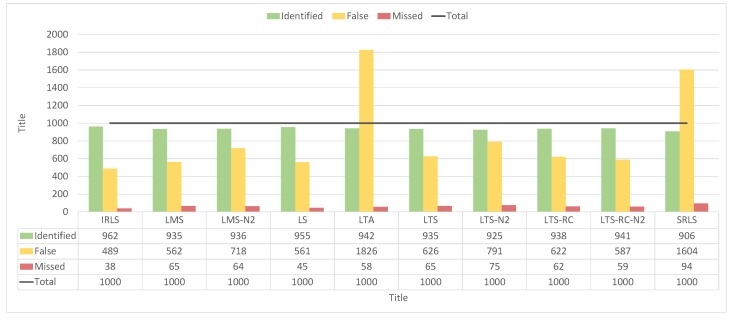
Number of identified false positives and undetected outliers for each estimator in the 400 scenarios with outliers of larger magnitude.

By counting the detected false positives and undetected outliers, all estimators pointed out most of the blunders, as can we see from Figure 18. For 1000 outliers, the values ranged from 906–962 for detection. As the detection of outliers is almost optimal, it remains to compare the amount of false positives, where IRLS, LS, LMS, and LTS-RC-N2 had the lowest false positive values, ranging from 489–587.

Table 3 presents the mean distance of solutions from the original co-ordinates. LS presented an unsatisfactory estimate for large outliers. The solution deteriorated, even with only one outlier of great magnitude. As we added more blunders to the scenarios, LS became one of the worst solutions for direct parameter estimation. LTA and SRLS also showed bad estimates, whereas most estimators presented similar, more satisfactory results, proving greater insensitivity to the outliers.

In general, for outlier identification, the LS, IRLS, LMS, or LTS-RC-N2 methods had better detection. Regarding the computational cost of these solutions, LS is the best choice because it has its own iterative method.

However, for the final estimate of the co-ordinates, the REs presented more satisfactory estimates. This shows the sensitivity of LS to outliers of great magnitude and the strength of robust methods.

Table 4 presents the best estimator, concerning the application and scenario of errors.

## 6. Conclusions and Future Works

Geodetic networks are the basis for mapping, geoinformation, land cadastre, and other location-based services. They play an important role in society in infrastructural works that depend on accurate control points. This work tested the strategy of using Robust Estimators (REs) in geodetic network adjustment and for detection of outliers.

Several REs were tested. A metaheuristic optimization was conducted for the LTS, LTS-RC, LMS, SRLS, and LTA estimators, whereas, for the IRLS, an iterative process was handled.

The two main contributions of the research were successfully demonstrated: (1) the Independent Vortices Search (IVS) overcame the VS, MVS, HVS, and ABC in all aspects; (2) we performed a deep investigation of several REs, separating the analysis scenarios into no outliers, small outliers, and outliers of great magnitude. This led to the classifications in Table 4, something not explored in the literature before.

In addition, other minor contributions were also presented. One of them was the search space proposed for applying IVS using Equation (27), which is valid not only for geodetic networks, but for any problem based on the Gauss–Markov model. Furthermore, a more detailed analysis of the results, obtained with Equations (7) and (18), was given in Section 5.2, showing which equation is more appropriate in each case.

The experiments with the geodetic network were all performed by applying the IVS, built on the Modified Vortex Search. IVS works with the vortices independently, which can also facilitate the parallelization of procedures which require high performance. This includes exploring more complex problems or even larger geodetic networks.

In the experiments of quality control in the geodetic networks, in situations with small outliers or seeking outlier identification, we do not recommend the LS method. In these cases, according to the experiments, it is better to work with IRLS, or, for a better identification at a cost of more false positives, LMS, LTS, or LTS-RC-N2 can achieve better results. Even though LS detects fewer outliers in these scenarios, it remains as the best estimator for the parameters, as the co-ordinates remain closer to their true values.

For estimating the co-ordinates in scenarios with large-scale outliers, REs present a more satisfactory estimate than LS. This showed the sensitivity of LS to outliers of high magnitude and the strength of the robust methods.

Although the computational cost of REs that use MHs is greater than the classical techniques, it is not possible to establish a cost-benefit at the moment. For this, it is first necessary to consider a minimum amount of FE to get good results, based on the RE and the network dimension. However, a disadvantage of this technique is that is it not possible to estimate the precision of the points. To achieve that, we need an LS estimation after removing the outliers from the observation vector.

Future works can focus on a scalability study and the parallelization of the IVS to achieve better performance. In robust estimation, other strategies can also be studied, such as testing new constraints to REs or checking other REs not contemplated by this work.

## Figures and Tables

**Table 1:** Packages containing 100 error scenarios each.

Package	Number of Simultaneous Outliers per Scenario	Magnitude of Outliers (*σ*)
1	0	-
2	1	[3,6)
3		[6,12]
4	2	[3,6)
5		[6,12]
6	3	[3,6)
7		[6,12]
8	4	[3,6)
9		[6,12]

**Table 2:** Mean distance of the solutions for each estimator, organized in the scenarios of 1–4 concurrent small outliers.

	LS (No Outliers)	LS	LMS	LTS	LTS-RC	SRLS	IRLS	LTS-RC-N2	LMS-N2	LTS-N2	LTA
1 outlier	0.014	0.017	0.023	0.024	0.024	0.026	0.024	0.021	0.023	0.024	0.029
2 outliers	0.014	0.020	0.025	0.027	0.027	0.028	0.027	0.026	0.025	0.027	0.032
3 outliers	0.014	0.022	0.027	0.027	0.027	0.030	0.025	0.027	0.028	0.028	0.035
4 outliers	0.014	0.025	0.029	0.029	0.030	0.031	0.024	0.030	0.029	0.031	0.036

**Table 3:** Mean distance of the solutions for each estimator, organized in the scenarios of 1–4 concurrent large outliers.

	LS (No Outliers)	LS	LMS	LTS	LTS-RC	SRLS	IRLS	LTS-RC-N2	LMS-N2	LTS-N2	LTA
1 outlier	0.014	0.023	0.022	0.023	0.023	0.030	0.023	0.023	0.025	0.025	0.037
2 outliers	0.014	0.031	0.025	0.027	0.027	0.036	0.026	0.027	0.025	0.028	0.039
3 outliers	0.014	0.037	0.024	0.028	0.028	0.040	0.028	0.028	0.029	0.031	0.044
4 outliers	0.014	0.045	0.036	0.031	0.031	0.043	0.032	0.031	0.034	0.036	0.051
Mean	0.014	0.034	0.027	0.027	0.027	0.037	0.027	0.027	0.028	0.030	0.043

**Table 4:** Estimators with best results, according to the demand and scenario. (*) indicates a higher false positives cost.

	Detection	Estimation
No outlier	-	LS
Small magnitude outliers	IRLS, LMS *, LTS *, LTS-RC-N2 *	LS
Large magnitude outliers	LS, IRLS, LMS, LTS-RC-N2	IRLS, LMS, LTS, LTS-RC, LTS-RC-N2

## References

[1] Ghilani C.D. (2010). Adjustment Computations.

[2] Leick A., Rapoport L., Tatarnikov D. (2015). GPS Satellite Surveying.

[3] De Guidi G., Brighenti F., Carnemolla F., Imposa S., Marchese S.A., Palano M., Scudero S., Vecchio A. (2018). The unstable eastern flank of Mt. Etna volcano (Italy): First results of a GNSS-based network at its southeastern edge. J. Volcanol. Geotherm. Res..

[4] Mahapatra P.S., Samiei-Esfahany S., Hanssen R.F. (2015). Geodetic Network Design for InSAR. IEEE Trans. Geosci. Remote. Sens..

[5] Hofmann-Wellenhof B., Lichtenegger H., Wasle E. (2008). GNSS—Global Navigation Satellite Systems.

[6] Teunissen P.J.G. (2018). Distributional theory for the DIA method. J. Geod..

[7] Lehmann R. (2013). On the formulation of the alternative hypothesis for geodetic outlier detection. J. Geod..

[8] Klein I., Matsuoka M.T., Guzatto M.P., Nievinski F.G., Veronez M.R., Rofatto V.F. (2018). A new relationship between the quality criteria for geodetic networks. J. Geod..

[9] Koch K.R. (1999). Parameter Estimation and Hypothesis Testing in Linear Models.

[10] Teunissen P.J.G. (2003). Adjustment Theory: An Introduction.

[11] Huber P.J., Ronchetti E.M. (2009). Robust Statistics.

[12] Tadjudin S., Landgrebe D. (2000). Robust parameter estimation for mixture model. IEEE Trans. Geosci. Remote. Sens..

[13] Wang Y., Zhu X.X. (2016). Robust Estimators for Multipass SAR Interferometry. IEEE Trans. Geosci. Remote. Sens..

[14] Wiśniewski Z. (2010). Msplit(q) estimation: estimation of parameters in a multi split functional model of geodetic observations. J. Geod..

[15] Baselga S. (2007). Global Optimization Solution of Robust Estimation. J. Surv. Eng..

[16] Baselga S., Garcia-Asenjo L. (2008). GNSS Differential Positioning by Robust Estimation. J. Surv. Eng..

[17] Baselga S., Garcia-Asenjo L. (2008). Global robust estimation and its application to GPS positioning. Comput. Math. Appl..

[18] Yetkin M., Berber M. (2012). Application of the Sign Constrained Robust Least Squares Method to Surveying Networks. J. Surv. Eng..

[19] Yetkin M., Berber M. (2014). Implementation of robust estimation in GPS networks using the Artificial Bee Colony algorithm. Earth Sci. Inform..

[20] Koch I.É., Veronez M.R., da Silva R.M., Klein I., Matsuoka M.T., Gonzaga L., Larocca A.P.C. (2017). Least trimmed squares estimator with redundancy constraint for outlier detection in GNSS networks. Expert Syst. Appl..

[21] Yetkin M. (2018). Application of robust estimation in geodesy using the harmony search algorithm. J. Spat. Sci..

[22] Kennedy J., Eberhart R. Particle swarm optimization. Proceedings of the ICNN’95—International Conference on Neural Networks.

[23] Karaboga D. (2005). An Idea Based on Honey Bee Swarm for Numerical Optimization.

[24] Karaboga D., Basturk B. (2007). A powerful and efficient algorithm for numerical function optimization: Artificial bee colony (ABC) algorithm. J. Glob. Optim..

[25] Karaboga D., Akay B. (2009). A comparative study of Artificial Bee Colony algorithm. Appl. Math. Comput..

[26] Dorigo M., Birattari M., Stutzle T. (2006). Ant Colony Optimization. IEEE Comput. Intell. Mag..

[27] Hussain K., Mohd Salleh M.N., Cheng S., Shi Y. (2018). Metaheuristic research: A comprehensive survey. Artif. Intell. Rev..

[28] Baarda W. (1968). A Testing Procedure for Use in Geodetic Networks.

[29] Rofatto V.F., Matsuoka M.T., Klein I., Veronez M.R., Bonimani M.L., Lehmann R. (2018). A half-century of Baarda’s concept of reliability: A review, new perspectives, and applications. Surv. Rev..

[30] Nakamura R.Y.M., Garcia Fonseca L.M., Dos Santos J.A., Da S Torres R., Yang X.-S., Papa Papa J. (2014). Nature-Inspired Framework for Hyperspectral Band Selection. IEEE Trans. Geosci. Remote. Sens..

[31] Suresh S., Lal S., Chen C., Celik T. (2018). Multispectral Satellite Image Denoising via Adaptive Cuckoo Search-Based Wiener Filter. IEEE Trans. Geosci. Remote. Sens..

[32] Dogan B., Ölmez T. (2015). A new metaheuristic for numerical function optimization: Vortex Search algorithm. Inf. Sci..

[33] Dogan B. (2016). A Modified Vortex Search Algorithm for Numerical Function Optimization. Int. J. Artif. Intell. Appl..

[34] Rousseeuw P. (1984). Least Median of Squares Regression. J. Am. Stat. Assoc..

[35] Xu P. (2005). Sign-constrained robust least squares, subjective breakdown point and the effect of weights of observations on robustness. J. Geod..

[36] Tableman M. (1994). The asymptotics of the least trimmed absolute deviations (LTAD) estimator. Stat. Probab. Lett..

[37] Guo J., Ou J., Wang H. (2010). Robust estimation for correlated observations: Two local sensitivity-based downweighting strategies. J. Geod..

[38] Rousseeuw P.J., Leroy A.M. (1987). Robust Regression and Outlier Detection.

[39] Hekimoglu S., Erenoglu R.C., Sanli D.U., Erdogan B. (2011). Detecting Configuration Weaknesses in Geodetic Networks. Surv. Rev..

[40] Rousseeuw P.J., Croux C. (1993). Alternatives to the median absolute deviation. J. Am. Stat. Assoc..

[41] Klein I., Matsuoka M.T., Guzatto M.P., de Souza S.F., Veronez M.R. (2015). On evaluation of different methods for quality control of correlated observations. Surv. Rev..

[42] Rofatto V.F., Matsuoka M.T., Klein I. (2018). Design of Geodetic Networks Based on Outlier Identification Criteria: An Example Applied to the Leveling Network. Bol. CiÊNcias GeodÉSicas.

[43] Knight N.L., Wang J. (2009). A Comparison of Outlier Detection Procedures and Robust Estimation Methods in GPS Positioning. J. Navig..

[44] Förstner W. (1987). Reliability analysis of parameter estimation in linear models with applications to mensuration problems in computer vision. Comput. Vis. Graph. Image Process..

[45] Lee C., Bethel J. (2001). Georegistration of airborne hyperspectral image data. IEEE Trans. Geosci. Remote. Sens..

[46] Rellier G., Descombes X., Falzon F., Zerubia J. (2004). Texture feature analysis using a gauss-Markov model in hyperspectral image classification. IEEE Trans. Geosci. Remote. Sens..

[47] Yu J., Lin Y., Wang B., Ye Q., Cai J. (2019). An Advanced Outlier Detected Total Least-Squares Algorithm for 3D Point Clouds Registration. IEEE Trans. Geosci. Remote. Sens..

[48] Google Earth. https://earth.google.com/web/.

[49] Wang Z., Wu G., Wan Z. (2017). A novel hybrid vortex search and artificial bee colony algorithm for numerical optimization problems. Wuhan Univ. J. Nat. Sci..

[50] Mernik M., Liu S.H., Karaboga D., Črepinšek M. (2015). On clarifying misconceptions when comparing variants of the Artificial Bee Colony Algorithm by offering a new implementation. Inf. Sci..

